# Effects of acute moderate-intensity aerobic exercise on cognitive function in E-athletes: A randomized controlled trial

**DOI:** 10.1097/MD.0000000000035108

**Published:** 2023-10-06

**Authors:** Weichao Zhang, Xiaoqiang Wang, Xun Li, Hongqiao Yan, Yuanyuan Song, Xinying Li, Wenhua Zhang, Guoao Ma

**Affiliations:** a Faculty of Postgraduate Education, Shandong Sport University, Jinan City, China; b College of Sports and Health, Shandong Sport University, Jinan City, China; c Department of E-sports, Shandong Sport University, Jinan City, China.

**Keywords:** acute aerobic exercise, cognition, eSports, reaction time, time perception

## Abstract

**Background:**

E-sports require athletes to have high-speed reflexes and excellent memory skills. Whereas a single session of aerobic exercise has been shown to improve cognitive function, this paper aims is to investigate the effects of acute moderate-intensity aerobic exercise on the cognitive function of e-sports players and its time-course characteristics.

**Methods:**

Thirty-four E-athletes were divided into 2 groups according to a random number table method, and 2 trials in a quiet physical fitness gym. The duration of each trial was approximately 1 hour. In the first trial: exercise group (64–76% of maximum heart rate for 30 minutes power cycling) and control group, cognitive function was tested, and results were automatically recorded before, immediately after, and 30 minutes after exercise using the human benchmark website (https://humanbenchmark.com). The second trial crossed and swapped the interventions of the 2 groups, and the other test protocols were the same as the first.

**Results:**

In both trials, the exercise intervention group showed significant improvements in speed accuracy (*P* < .001, Cohen’s *d* = 1.406, 95% CI: 0.717–2.072; *P* = .005, Cohen’s *d* = 0.782, 95% CI: 0.227–1.319), visual memory (*P* < .001, Cohen’s *d* = 1.416, 95% CI: 0.725–2.086; *P* = .015, Cohen’s *d* = 0.662, 95% CI: 0.127–1.181), and reaction time (*P* < .001, Cohen’s *d* = 1.265, 95% CI: 0.610–1.898; *P*<.001, Cohen’s *d* = 0.979, 95% CI: 0.386–1.551) immediately after exercise compared to baseline. The exercise intervention group also showed significant improvement in speed accuracy 30 minutes after exercise compared to baseline (*P* = .002 Cohen’s *d* = 0.869, 95% CI: 0.298–1.421; *P* = .009, Cohen’s *d* = 0.722, 95% CI: 0.177–1.249). In the first trial, the exercise intervention group showed significant improvements in visual memory and reaction time immediately after exercise compared to the control group (*P* = .013, Cohen’s *d* = 0.904, 95% CI: 0.190–1.605; *P* = .027, Cohen’s *d* = 0.796, 95% CI: 0.090–1.490). The exercise intervention group also showed significant improvement in reaction time 30 minutes after exercise compared to baseline (*P* = .009, Cohen’s *d* = 0.719, 95% CI: 0.174–1.246). There was no effect of exercise on sequence memory or the chimp test in both trials (*P* > .05). Sequence effect analysis showed no influence on the order of the exercise intervention in both trials (*P* = .912; *P* = .111; *P* = .226).

**Conclusion:**

Acute moderate-intensity aerobic exercise significantly enhanced the speed accuracy, visual reaction time, and instantaneous memory of eSports players, and the effect could be extended up to 30 minutes after exercise.

## 1. Introduction

E-sports have been attracting more and more attention, with the number of spectators reaching 495 million and the revenue reaching approximately $1.1 billion in 2020.^[1]^ According to the International Federation of eSports, eSports is officially accepted as a sport in more than 60 countries. In the 2022 Asian Games in China, eSports will appear as an official sport. Scientists have highlighted the scientific principles of eSports as a demonstration of the benefits of enhancing cognitive abilities to improve athletic performance.^[2]^ The popularity of e-sports has also led to a diversification of e-sports events, including action, adventure, sports, and real-time strategy games.^[3]^ Each type of game has different demands on players’ skills, but most require precision and reaction speed, with milliseconds often deciding the outcome. In addition, e-sports requires higher visual demands and eye health than traditional sports, so e-sports players require excellent visual memory and rapid response capabilities.^[1,3]^ So, professional eSports athletes spend hours each day playing the video games they excel at to improve specific gaming skills, including gamepad and keyboard manipulation, game knowledge, and strategy and tactics. Research has found that e-sports players train for an average of 5.5 to 10 hours daily, with continuous training in a seated position lasting up to 3 hours or even longer.^[4]^ Elite athletes spend more than ten hours daily practicing dynamic and repetitive movements with a mouse and keyboard, executing up to 400 fine movements per minute.^[5]^ These prolonged states of cognitive, physical, and mental strain can promote the development of adverse health-related side effects.^[6]^ Yi^[7]^ once pointed out the “ten sins of e-sports, including “stiff and twisted fingers, muscle damage, chronic diseases, and cardiovascular diseases.”

The link between physical activity and the brain has been of interest for some time, with most studies examining the effects of low to moderate-intensity aerobic exercise and brain function. Aerobic exercise has also been reported to increase brain endothelial cell proliferation and angiogenesis.^[8,9]^ Research has shown that exercise is a potent stimulus for brain plasticity and has been shown to enhance hippocampal cell proliferation and improve hippocampal memory tasks.^[10]^ Visual reaction time, visual memory, speed accuracy, etc. are the most basic cognitive tasks. Visual reaction time refers to the time interval between the appearance of a stimulus and the body’s response. Studies have found that gamers generally have better visual reaction times than non-gamers.^[1,11]^ Visual memory, such as sequence and instant memory, is also crucial in some e-sports games. Professional e-sports players have been reported to have better memory skills in this area.^[1]^ Reaction time and visual memory mainly reflect the flexibility of brain neural activity and, to some extent, reflect cognitive abilities such as adaptability and attention features.^[12]^ A critical factor in cognitive performance is maintaining sustained attention or vigilance for a given stimulus or task. And a decrease in vigilance can result in slower reaction times or increased error rates^[13]^; this situation is very serious for eSports players.

Acute aerobic exercise, also known as single-session exercise, lasting 10 to 60 minutes, is a type of exercise that uses aerobic metabolism to provide energy. It has been shown to affect cognitive function positively.^[14]^ A meta-analysis of acute aerobic exercise found widespread beneficial effects on cognitive abilities such as reaction time and accuracy.^[15]^ Brain-derived neurotrophic factor (BDNF) plays a vital role in neuronal remodeling, synaptic plasticity, and neurotransmitter release, and acute aerobic exercise can enhance BDNF in the hippocampus of rodents.^[16]^ A systematic review found that acute aerobic exercise enhances neural plasticity and improves executive function, attention, and memory, partly through the upregulation of neurotrophic factors such as BDNF.^[17]^

Numerous pieces of evidence demonstrate the benefits of acute aerobic exercise, and there are numerous scientific studies on acute aerobic exercise interventions. However, although all their studies aimed to explore the effects of acute exercise on cognitive function in the general population, few studies have examined the effects of acute moderate-intensity aerobic exercise on the cognitive function of professional E-athletes. Most studies were limited to observing the immediate effects of acute aerobic exercise. They did not further explore the time-course characteristics of acute exercise.^[18,19]^ The most critical aspect of eSports players is the operational executive function. These goal-directed cognitive processes coordinate and regulate thought and action. Each eSports event lasts about 30 to 60 minutes, which requires eSports players to maintain good operational executive function throughout the process. Both cognitive function enhancement and physical training for this specialized population must have an optimal acute exercise dose and appropriate cognitive tasks. Only in this way can it be ensured that the eSports player’s executive cognitive function can be enhanced and that the competition state is not disturbed by the acute exercise intensity. ESports athletes need optimal cognitive, physical, and mental training to achieve the necessary cognitive and physical optimum and to counteract the general health problems caused by hours of training in front of the PC table.^[6]^ In previous studies on acute exercise, the study often conducted only 1 trial on the subject and then concluded the results.^[15,17,18]^ However, in some cases, there are errors in this testing, resulting in tests that do not conclude a 1-time exercise. However, recently there has been a new approach to the test protocol regarding 1-time exercise, which is to conduct another acute test after an interval and to cross-swap the intervention methods of the 2 groups of subjects, which can more accurately reflect the effect of acute exercise and thus exclude the error caused by the sequence effect.^[20]^ Therefore, in terms of the experimental method and the research objectives, this experiment has significantly improved and new exploration compared to previous studies, proving this study’s necessity.

In summary, does a single bout of moderate-intensity physical exercise positively affect e-sports players’ cognitive functions, such as reaction time, accuracy, and visual memory? What are the time course characteristics of the impact on these cognitive functions? This study used laboratory experiments to observe the effects of acute moderate-intensity aerobic exercise on the cognitive function of e-sports players and to explore the time course characteristics of these effects. This benefit will allow eSports players to maintain excellent cognitive executive function after exercise, contributing to higher performance levels in competition. Also, given the benefits of acute aerobic exercise, the results of this experiment will help e-sports coaches or personal physicians to target better training and recovery integration training programs for e-sports players.

We hypothesize that acute aerobic exercise intervention will improve e-sports players’ cognitive functions, such as reaction time, accuracy, and visual memory. The effects will exhibit specific time course characteristics.

## 2. Participant and methods

### 2.1. Participants

In October 2022, eligible E-athletes were openly recruited for the e-sports major at Shandong Sport University. Shandong Institute of Sports, the first higher education institution in China to offer an eSports undergraduate program, has high-quality E-athletes who are strictly different from ordinary healthy people. All subjects were E-athletes who had participated in relevant eSports tournaments and had previous extensive eSports real-world experience. The inclusion criteria are as follows:

1. Good physical health with no history of illness or family diseases;
2. Playing time exceeding 14 hours per week for 6 months;
3. Voluntary participation in the study and ability to complete training according to the requirements;
4. Right-handed.

The exclusion criteria are as follows:

1. Severe injury or unrecovered sports injury history;
2. Recent taken any drugs that have exciting or paralyzing effects on nerves;
3. Participation in similar experimental projects within 3 months;
4. Unwillingness to participate in the study;
5. Red-green color blindness.

Sample size estimation was conducted based on the results of the pre-experiment regarding the Sequence memory test, Chimp test, Aim trainer test, Visual memory, and Reaction time. Sequence memory test (points): Experimental group (pre = 8.25 ± 1.50, post = 11.25 ± 0.96), control group (pre = 8.25 ± 1.26, post = 9.00 ± 1.41). Chimp test (points): Experimental group (pre = 8.00 ± 2.16, post = 10.50 ± 1.00), control group (pre = 8.50 ± 1.29, post = 8.75 ± 0.96). Aim trainer test (ms): Experimental group (pre = 614.75 ± 41.99, post = 484.75 ± 39.54), control group (pre = 548.50 ± 42.63, post = 531.25 ± 39.69). Visual memory (points): Experimental group (pre = 9.75 ± 0.96, post = 12.00 ± 0.82), control group (pre = 10.50 ± 1.73, post = 11.50 ± 1.39). Reaction time (ms): Experimental group (pre = 252.00 ± 39.27, post = 231.75 ± 30.36), control group (pre = 243.00 ± 17.61, post = 238.00 ± 12.83). After conducting a repeated measures analysis of variance, the effect sizes were found to be η^2^_p_ = 0.786, 0.463, 0.635, 0.532, and 0.431 for speed accuracy, visual memory, and reaction time, respectively. Using the F tests function in G-power software, with control of Type I error rate at 0.05, Type II error rate at 0.20 or below, and statistical power of 80% or higher, the corresponding effect sizes were calculated as Cohen’s *d* = 1.916, Cohen’s *d* = 0.929, Cohen’s *d* = 1.319, Cohen’s *d* = 1.066, and Cohen’s *d* = 0.870. The required sample sizes were estimated as 12, 27, 17, 22, and 30, respectively. Considering sample loss, we ultimately recruited 40 participants, but 6 refused to participate and were excluded, leaving 34 participants.

The 34 participants were randomly divided into 2 groups of 17 people each. The randomization method is the random number table method, which uses a computer to generate a random number table containing 50 natural numbers in 10 rows and 10 columns, all randomized horizontally, vertically, or diagonally. First, 34 consecutive digits of a particular section were random selected from the random number table. Then the 34 subjects were numbered in the order of group entry and arranged in the order corresponding to the random numbers, specifying that those with odd corresponding random numbers entered Group 1 and those with even corresponding random numbers entered Group 2. Complete randomization of the groups can be achieved with this method.

This study was conducted by the Helsinki Declaration and was approved by the Ethics Committee of Sports Science (approval number: 2022007). The responsible person informed the participants of the study content and procedures verbally and in writing. All participants were given written informed consent before voluntary participation in the study and signed a paper consent form. Participants were free to withdraw from the study without further consequences.

### 2.2. Cognitive tasks

All cognitive tasks were tested using the Human Benchmark website (https://humanbenchmark.com), which automatically records relevant metrics data. The website can test subjects’ reaction time, speed accuracy, and memory ability. The test is not affected by equipment differences and is measured by mouse clicks only, with negligible measurement error. The Web site has been widely used in scientific research to measure simple cognitive tasks for e-sports players or general gamers.^[1]^

#### 2.2.1. Sequence memory test.

The sequence memory test assesses long-term memory. This study used sequence memory test from the Human Benchmark website (https://humanbenchmark.com/tests/sequence) to evaluate memory ability. Participants had to remember the sequence in which buttons lit up and press them in the same order. The length of the test increased as the level increased, and the test ended if an error occurred. The current level represented the final score.

#### 2.2.2. Chimp test.

The Chimp test evaluates long-term memory. This study used Chimp test from the Human Benchmark website (https://humanbenchmark.com/tests/chimp) to evaluate memory ability. At the beginning of the test, participants were asked to remember the location of white squares in numerical order and click on them in the same sequence. The test started with 4 numbers and increased by one number for each level. Participants lost one chance if an error occurred, and they had 3 chances. The final score was the level reached after 3 chances were used up.

#### 2.2.3. Aim trainer test.

The Aim trainer test evaluates speed and accuracy abilities. This study used Aim trainer test from the Human Benchmark website (https://humanbenchmark.com/tests/aim) to evaluate speed and accuracy abilities. At the beginning of the test, participants were asked to click on 30 targets on the screen as quickly and accurately as possible. The average time for each target was calculated in milliseconds (ms) at the end of the test.

#### 2.2.4. Visual memory test.

The Visual memory test evaluates memory ability. This study used Visual memory test from the Human Benchmark website (https://humanbenchmark.com/tests/memory) to evaluate short-term memory ability. At the beginning of the test, participants were asked to remember the location of a flashing white square. The difficulty increased with each level, and participants lost one chance if they made 3 mistakes at a certain level. They had a total of 3 chances, and the final score was the level reached after all 3 chances were used up.

#### 2.2.5. Reaction time test.

The Reaction time test evaluates visual reaction time. This study used Reaction time test from the Human Benchmark website (https://humanbenchmark.com/tests/reactiontime) to evaluate the visual reaction time. After the participant was ready, the test started, and they were asked to press a button immediately when a green light appeared on the screen. The test was conducted 5 times, and the average reaction time was calculated in milliseconds (ms).

### 2.3. Experimental tools

The main experimental equipment used during the exercise intervention included: Lode Corival Aerobic Power Bike (Lode B.V., Groningen, Netherlands) and Polar RS400 Heart Rate Telemetry (including bracelet and chest strap) (Polar Electro Oy, Oulu, Finland), which monitored in real time the heart rate changes of the subjects during pre- and post-exercise sessions. The cognitive task tests were all required to be completed by the subjects using the specified Lenovo Legion R720 computer (Lenovo Group Limited, Beijing, China).

### 2.4. Exercise program

Participants were randomly assigned to Group 1 and Group 2, and the test was divided into 2 experiments. In the first experiment, Group 1 used the control method (Group C), and Group 2 used the exercise intervention method (Group E). In the second experiment, the 2 groups were crossed over, meaning that Group 1 used the exercise intervention method (Group E) and Group 2 used the control method (Group C). The exercise intervention method first used the power bicycle for 30 minutes (5 minutes for warm-up activity). The exercise intensity was based on the latest rating standards for exercise intensity for healthy adults from the American College of Sports Medicine. The moderate-intensity exercise load was set to 64% to 76% of the individual’s maximum heart rate, with maximum heart rate (HR_max_) = 207 - 0.7 × age. Each subject was required to reach the target heart rate range set during exercise and maintain it for 25 minutes, followed by 30 minutes of rest. The control method used a blank control with no exercise measures, sitting still throughout to keep the heart rate steady, and completing the cognitive task test at a quiet heart rate.

The most commonly used form of exercise for acute aerobic exercise is power cycling, which has the advantage of being able to control heart rate by pedaling speed as a way to achieve moderate exercise intensity. The crossover of the intervention modality between the 2 groups of subjects in both trials was to verify the presence of a sequential effect and, thus, to more accurately verify the effect of acute moderate-intensity aerobic exercise on cognitive function.

### 2.5. Experimental procedure

First, inform the subjects of the experimental procedure and obtain their informed consent. To overcome the effects of subject response, inform the subjects before the experiment begins that this experiment is designed to measure the impact of a 1-time exercise on cognitive function. Then, have the exercise group subjects wear the heart rate telemetry device. In the first experiment: the first index test is performed before exercise, recorded as Time 0, followed by 30 minutes of exercise intervention or control, and then the second index test is performed immediately after exercise, recorded as Time 30. Both groups rested in place for 30 minutes, and the third index test was performed immediately after the rest, recorded as Time 60. The first experiment ended. After a week’s interval, the second experiment was conducted; except for the intervention methods for each group being crossed over, the other procedures were the same as in the first test. Both experiments were conducted in the laboratory of the Shandong Institute of Physical Education, and the experiments were started simultaneously. The experimental environment was kept quiet, ventilated and, at a suitable temperature. The duration of a single test was approximately 1 hour.

### 2.6. Statistical analysis

#### 2.6.1. Descriptive statistics.

We used descriptive statistics for the baseline characteristics of the subjects, including the mean and standard deviation of age, sex ratio, height, weight, BMI, and age at play.

#### 2.6.2. Repeated measures ANOVA.

Statistical analysis was performed using statistical software SPSS 26.0 (IBM, Chicago, IL) and GraphPad Prism 9.0 software (GraphPad Software, San Diego, CA). The Shapiro–Wilk test was used to test the normal distribution of each continuous variable. Descriptive mean and standard deviation statistics were used for variables that followed a normal distribution, while the median and interquartile range were used for non-normally distributed data. A repeated measure two-way analysis of variance was used to determine the effects of exercise intervention and control on each outcome variable.

#### 2.6.3. Multiple comparison correction.

A Bonferroni-adjusted post hoc analysis was conducted when time-group interaction was detected. The effect size of the main effect and interaction was expressed using partial eta squared (η^2^_p_). The threshold for partial eta squared was: small if 0.01–0.06, medium if 0.06–0.14, and large if >0.14. Cohen’s d was used to indicate the effect size of post hoc comparisons. The threshold for Cohen’s *d* was: negligible if <0.20, small if 0.21–0.50, medium if 0.51–0.80, and large if >0.81.

#### 2.6.4. Sequential effects test.

Independent samples *t* test and Mann–Whitney test were used to evaluate whether there was a sequence effect between exercise and control.

### 2.7. Experimental flow chart

**Figure:**
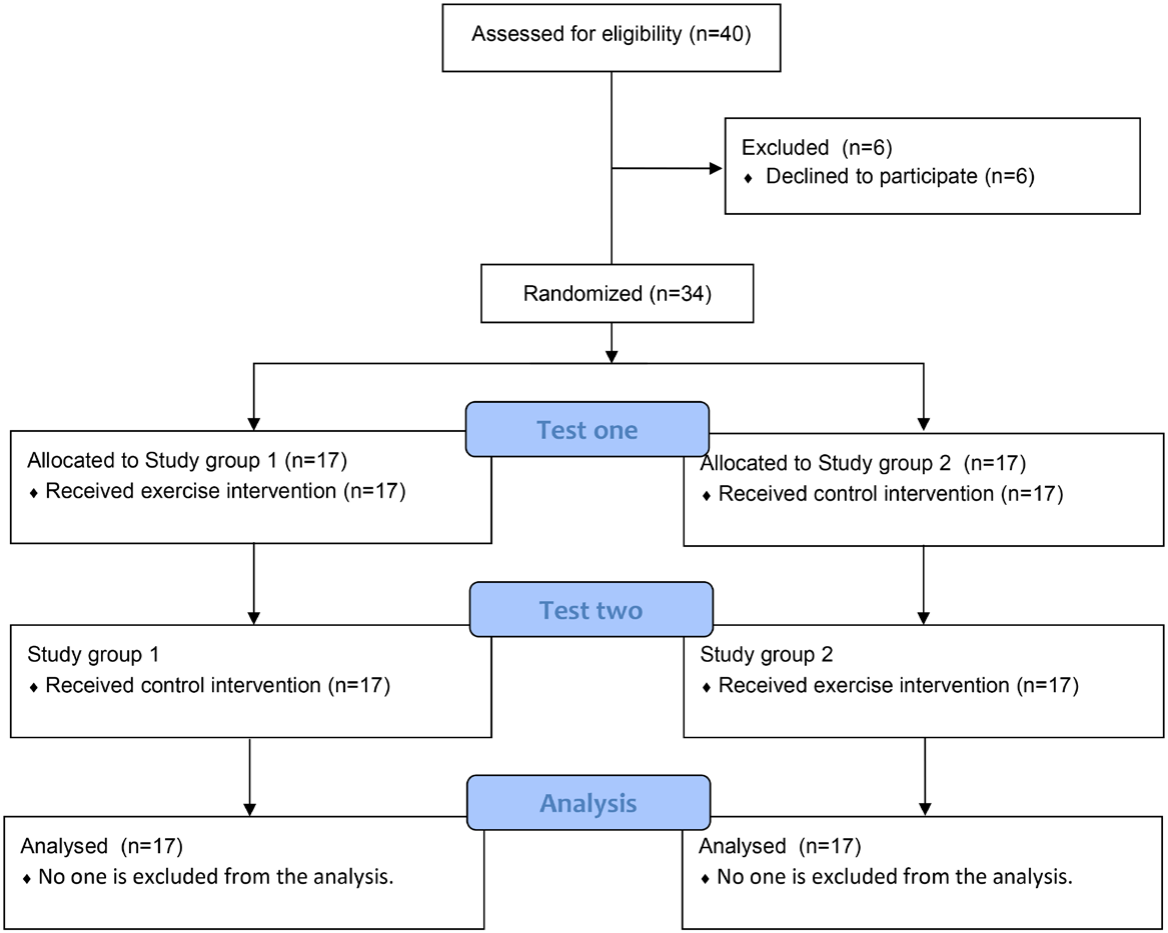


## 3. Results

### 3.1. Basic information of participants

The Shapiro–Wilk test showed that all dependent variables were normally distributed (*P* > .05). An independent samples *t* test was conducted to test for differences in the essential characteristics of the subjects between the 2 groups. As shown in Table 1, there were no significant differences between Group 1 and Group 2 regarding age, height, weight, BMI, or gaming experience (*P* > .05). The gender ratio was also similar between the 2 groups.

**Table 1:** Baseline demographics of the study population.

	Study group 1 (n = 17)	Study group 2 (n = 17)	*P*
Age, yr	18.59 ± 1.00	18.24 ± 0.44	.347
Female sex, %	17.65	17.65	—
Height, m	1.76 ± 0.07	1.75 ± 0.07	.571
Body weight, kg	70.24 ± 12.20	63.29 ± 12.15	.106
BMI, kg/m^2^	22.54 ± 3.40	20.58 ± 2.97	.083
Game age, yr	7.24 ± 3.19	7.53 ± 3.94	.812

### 3.2. Results of the first trial

According to Table 2, there was no interaction effect of time and group on the sequence memory and chimpanzee tests for Groups C and E (*P* = .607, η^2^_p_ = 0.032; *P* = .158, η^2^_p_ = 0.112), and there were no group or time effects (*P* > .05). The speed-accuracy test had no interaction effect (*P* = .247, η^2^_p_ = 0.086). However, a time effect was observed, where the speed-accuracy of Group E was significantly better immediately after exercise than before (*P* < .001, Cohen’s *d* = 1.406, 95% CI: 0.717–2.072), and 30 minutes after exercise (*P* = .002 Cohen’s *d* = 0.869, 95% CI: 0.298–1.421). The visual memory test had no interaction effect (*P* = .080, η^2^_p_ = 0.150). However, there were time and group effects, where the visual memory of Group E was significantly better immediately after exercise than before (*P* < .001, Cohen’s *d* = 1.416, 95% CI: 0.725–2.086) and significantly better than Group C at the same time (*P* = .013, Cohen’s *d* = 0.904, 95% CI: 0.190–1.605). In the reaction time test, there was an interaction effect of time and group (*P* = .031, η^2^_p_ = 0.138), where the reaction time of Group E was significantly faster immediately after exercise than before (*P* < .001, Cohen’s *d* = 1.265, 95% CI: 0.610–1.898), 30 minutes after exercise (*P* = .009, Cohen’s *d* = 0.719, 95% CI: 0.174–1.246), and significantly faster than Group C at the same time(*P* = .027, Cohen’s *d* = 0.796, 95% CI: 0.090–1.490).

**Table 2:** Analysis of variability of relevant indicators in the first trial.

First trial	Control group (n = 17)			Exercise group (n = 17)			Time × group		Post hoc	
	Time_0_	Time_30_	Time_60_	Time_0_	Time_30_	Time_60_	*P*	η^2^_p_	*P*	Cohen’s *d*
Sequence memory test (points)	9.82 ± 3.26	8.71 ± 2.62	8.76 ± 1.82	9.94 ± 2.86	9.76 ± 3.83	9.94 ± 4.24	.607	0.032	—	—
95% CI	8.15, 11.50	7.36, 10.05	7.83, 9.70	8.47, 11.41	7.79, 11.74	7.76, 12.12				
Chimp test (points)	9.82 ± 2.13	10.71 ± 1.21	10.47 ± 1.23	10.82 ± 1.29	10.47 ± 1.18	10.65 ± 1.32	.158	0.112	—	—
95% CI	8.73, 10.92	10.08, 11.33	9.84, 11.10	10.16, 11.48	9.86, 11.08	9.97, 11.33				
Aim trainer test (ms)	530.35 ± 75.94	518.71 ± 73.86	514.18 ± 59.69	533.29 ± 91.43*†	483.53 ± 79.73	499.59 ± 86.70	.247	0.086	*a* < 0.001 *b* = 0.002	*a* = 1.406 *b* = 0.869
95% CI	491.31, 569.40	480.73, 556.68	483.49, 544.87	486.29, 580.30	442.53, 524.52	455.01, 544.17				*a*(0.717, 2.072) *b*(0.298, 1.421)
Visual memory test (points)	9.88 ± 1.69	10.41 ± 1.33	10.29 ± 1.53	10.12 ± 1.58*	11.71 ± 1.53‡	10.88 ± 2.74	.080	0.150	*a* < 0.001 *c* = 0.013	*a* = 1.416 *c* = 0.904
95% CI	9.01, 10.75	9.73, 11.09	9.51, 11.08	9.31, 10.93	10.92, 12.49	9.48, 12.29				*a*(0.725, 2.086) *c*(0.190, 1.605)
Reaction time test (ms)	251.00 ± 34.45	238.41 ± 35.15	238.65 ± 35.32	250.82 ± 57.69*†	212.47 ± 29.84‡	231.41 ± 53.96	.031	0.138	*a* < 0.001 *b* = 0.009 *c* = 0.027	*a* = 1.265 *b* = 0.719 *c* = 0.796
95% CI	233.29, 268.71	220.34, 256.48	220.49, 256.81	221.16, 280.49	197.13, 227.81	203.67, 259.16				*a*(0.610, 1.898) *b*(0.174, 1.246) *c*(0.090, 1.490)

* Significant difference compared with Time_30_ in the Exercise group.

† Significant difference compared with Time_60_ in the Exercise group.

‡ Significant difference compared with the Control group at the same time.

### 3.3. Results of the second trial

According to Table 3, no significant interaction effects were found for the sequence memory and chimpanzee tests between the C and E groups in terms of time and group (*P* = .964, η^2^_p_ = 0.002; *P* = .879, η^2^_p_ = 0.008), and there were no significant time or group effects (*P* > .05). There was no significant interaction effect in the speed-accuracy test (*P* = .419, η^2^_p_ = 0.055), but a significant time effect was observed where the E group showed significantly better speed-accuracy immediately after exercise compared to before (*P* = .005, Cohen’s *d* = 0.782, 95% CI: 0.227–1.319); and 30 minutes after exercise (*P* = .009, Cohen’s *d* = 0.722, 95% CI: 0.177–1.249). The visual memory test found no significant interaction effect (*P* = .080, η^2^_p_ = 0.150). However, a significant time effect was observed where the E group showed significantly better visual memory immediately after exercise than before (*P* = .015, Cohen’s *d* = 0.662, 95% CI: 0.127–1.181). In the reaction time test, there was a significant interaction effect between time and group (*P* = .036, η^2^_p_ = 0.194), where the E group showed significantly faster reaction time immediately after exercise compared to before (*P*<.001, Cohen’s *d* = 0.979, 95% CI: 0.386–1.551), and immediately after exercise compared to 30 minutes after exercise (*P* = .007, Cohen’s *d* = 0.750, 95% CI: 0.196–1.282).

**Table 3:** Analysis of variability of relevant indicators in the second trial.

Second trial	Control group (n = 17)			Exercise group (n = 17)			Time × group		Post hoc	
	Time_0_	Time_30_	Time_60_	Time_0_	Time_30_	Time_60_	*P*	η^2^_p_	*P*	Cohen’s *d*
Sequence memory test (points)	10.00 ± 2.53	9.47 ± 2.50	9.65 ± 3.66	11.06 ± 3.11	10.24 ± 3.47	10.76 ± 3.17	.964	0.002	—	—
95% CI	8.70, 11.30	8.18, 10.76	7.77, 11.53	9.46, 12.66	8.45, 12.02	9.13, 12.40				
Chimp test (points)	10.29 ± 1.21	10.88 ± 1.41	10.53 ± 1.18	10.65 ± 1.46	11.06 ± 1.85	10.65 ± 1.54	.879	0.008	—	—
95% CI	9.67, 10.92	10.16, 11.61	9.92, 11.14	9.90, 11.40	10.11, 12.01	9.86, 11.44				
Aim trainer test (ms)	547.76 ± 66.71	526.59 ± 71.83	522.18 ± 62.88	526.41 ± 86.20*†	486.12 ± 68.76	495.24 ± 66.48	.419	0.055	*a* = 0.005 *b* = 0.009	*a* = 0.782 *b* = 0.722
95% CI	513.47, 582.06	489.66, 563.52	489.85, 554.51	482.09, 570.73	450.76, 521.47	461.06, 529.41				*a*(0.227,1.319) *b*(0.177,1.249)
Visual memory test (points)	10.06 ± 1.30	10.88 ± 2.45	10.53 ± 1.70	10.24 ± 1.25*	11.41 ± 1.54	11.18 ± 1.70	.407	0.022	*a* = 0.015	*a* = 0.662
95% CI	9.39, 10.73	9.62, 12.14	9.66, 11.40	9.59, 10.88	10.62, 12.21	10.30, 12.05				*a*(0.127,1.181)
Reaction time test (ms)	260.41 ± 47.37	242.88 ± 36.47	241.12 ± 43.23	257.06 ± 49.25*	225.71 ± 34.70†	237.76 ± 33.82	.036	0.194	*a* < 0.001 *b* = 0.007	*a* = 0.979 *b* = 0.750
95% CI	236.06, 284.76	224.13, 261.63	218.89, 263.34	231.74, 282.38	209.47, 241.94	220.38, 255.15				*a*(0.386, 1.551) *b*(0.196, 1.282)

* Significant difference compared with Time_30_ in the Exercise group.

† Significant difference compared with Time_60_ in the Exercise group.

### 3.4. Sequential effect analysis

**Figure 1.**
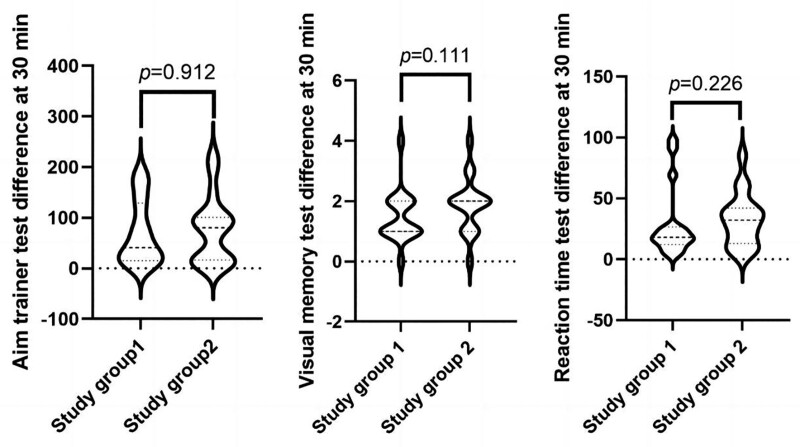
Comparison of inter-individual differences in each index after 30 min of exercise.

Further comparisons were made to determine if there were any order effects in the 2 experiments. In Group 1, the first experiment was the speed and accuracy test (X1), and the second experiment (cross-over) was the same test for the exercise group (X2). The difference was calculated as X = |X1 − X2|. In Group 2, the first experiment was the aiming response test for the exercise group (Y1), and the second experiment (cross-over) was the same test for the control group (Y2). The difference was calculated as Y = |Y1 − Y2|. The evaluation method for visual memory and reaction time tests was the same. The difference in results (X and Y) between Group 1 and Group 2 showed no statistically significant differences (*P* = .912 for the aiming response test, *P* = .111 for the visual memory test, *P* = .226 for the reaction time test), indicating that the intervention sequence did not affect the results, as shown in Figure 1.

## 4. Discussion

Our study aimed to investigate whether acute aerobic exercise benefits cognitive function in a specific occupational group of eSports players. As the best beneficiaries of cognitive executive function, eSports athletes urgently need an exercise training program that can rapidly improve their cognitive executive performance. At the same time, the unique nature of the eSports profession has led to a series of health problems in eSports athletes due to their sedentary lifestyle, including “stiff and twisted fingers, muscle damage, chronic disease, and cardiovascular disease.”^[7]^ Long-term gaming training also hurts the brain state of e-sports players, and retired professional e-sports players show more obvious neurological symptoms, such as dizziness, decreased concentration, and memory. A recent study showed that 40% of e-sports players still need physical exercise or daily activities for less than 60 minutes.^[4]^ Lack of exercise has become a significant cause of many chronic diseases. More and more evidence supports that exercise can promote neural development, and regular exercise can also have an anti-inflammatory effect and improve brain function.^[21,22]^ For e-sports players, exercise can alleviate various muscle and skeletal diseases caused by long-term training and improve brain function, thus enhancing cognitive performance in e-sports, achieving 2 goals at once.

### 4.1. Mechanisms of the benefits of exercise

There are currently several mechanisms regarding exercise-induced benefits, which include exercise increases microcirculation and mitochondrial function, which results in a significant ameliorative function for muscle,^[23,24]^ physical activity reduces a variety of inflammatory responses through immunomodulatory effects,^[25]^ exercise effectively reduces muscle fibrosis,^[26]^ exercise induces a reduction in endoplasmic reticulum stress, which prevents and prevents muscle degeneration and expression of malignant factors,^[27,28]^ and another point is that exercise enhances cardiorespiratory adaptations, which are significantly negatively correlated with all-cause mortality.^[29]^

As “lack of time” is often cited as the most common barrier to increasing habitual physical activity, recent years have seen a renewed interest in “time-efficient” physical activity,^[30]^ typically short, high-intensity exercise that triggers acute activation of signaling pathways^[31]^ and Cardiovascular, mitochondrial and metabolic adaptations^[32,33]^ are similar to those of more traditional low-intensity, endurance exercise programs. Interrupting prolonged sitting by light walking or simple resistance exercise is metabolically beneficial for people with type 2 diabetes,^[34]^ and short, repetitive (4-second) sprints may prevent metabolic damage associated with inactivity.^[35]^ The combination of increased physical activity and reduced sedentary behavior may lead to greater disease risk reduction.^[36]^ Regarding the effects of exercise on brain health and cognitive functioning, an emerging area in recent years,^[37,38]^ several studies have found that short bouts of exercise promote executive control of cognitive control functions^[39–41]^ and that short bouts of aerobic exercise of varying intensities have a positive impact on the production of selectivity in executive functioning.^[39]^

Acute aerobic exercise, also known as a 1-time exercise, lasts from 10 to 60 minutes and provides energy in a single session with aerobic metabolism, acute aerobic exercise.^[14]^ A systematic evaluation found that acute aerobic exercise enhanced neuroplasticity and enhanced human executive function, attention, and memory.^[17]^ In the present study, we found that the effects of acute aerobic exercise were different for different cognitive tasks and also that the enhancement of cognitive function by acute aerobic exercise persisted for some time. That is, there are temporal characteristics.

### 4.2. The effects of acute moderate-intensity aerobic exercise on different cognitive tasks vary

This study found that acute moderate-intensity aerobic exercise has varying effects on different cognitive tasks. Based on the results of the 2 experiments, exercise for 30 minutes can improve speed and accuracy, instant visual memory, and shorten reaction time. This is consistent with Peng and Zhou’s^[19]^ acute aerobic exercise test on female university students, which showed that 30 minutes of exercise could promote cognitive function. In the first experiment, the exercise group’s visual memory and visual reaction time was significantly better than the control group after 30 minutes of exercise. Early studies on the effects of acute aerobic exercise on simple cognitive tasks found that it had a promoting effect, mainly manifested in a decrease in reaction time and no change in accuracy.^[42]^ In addition, the improvement effect of exercise on cognitive function is also affected by exercise intensity. A study required participants to perform the acute aerobic exercise of different intensities and found that the choice reaction time of participants was significantly shortened. The degree of shortening of reaction time increased with the increased exercise intensity.^[43]^ Currently, there are 2 theories to explain why acute aerobic exercise can improve simple cognitive tasks. One is that exercise enhances sensory sensitivity, and the other is that exercise improves the efficiency of motor skills and speeds up key pressing. When participants perform cognitive task tests through a mouse or key, once an individual’s sensory sensitivity is improved, both the speed of receiving visual signals and the speed of executing clicking actions by hand muscles can be accelerated, thereby promoting cognitive performance. In a related study that included electromyography data, it was ultimately found that acute aerobic exercise had an impact on both sensory processing and action response, indicating that reaction time, speed and accuracy, and simple memory tasks can be achieved through 2 pathways of sensory processing and action response.^[44]^

The experiment results did not find a promotion effect of exercise on sequential memory tests and chimpanzee tests, which are tests of sequential memory ability. Tomporowski^[45]^ once pointed out that acute aerobic exercise does not affect memory encoding and retrieval, but some scholars believe that acute aerobic exercise promotes memory. This also indicates that the effects of acute aerobic exercise on memory may be influenced by the type, difficulty, and time of memory tasks, resulting in divergent research conclusions. The reason for this result in this article may be that during the indicator testing process, it was found that because there was no time limit for the sequence memory test and chimpanzee test, that is, the participants could subjectively choose to spend more time thinking and memorizing, which led to the fact that in the testing process of these 2 indicators, the time spent by each participant was significantly different, and those who spent more time got high scores. The effects of exercise may be offset by the time spent, which indicates that the results of these 2 indicators are influenced to a certain extent by time. However, overall, more scholars believe that acute aerobic exercise promotes memory. The standard research method is to let participants perform a memory task before and after exercise and then evaluate the memory content. The results show that the memory learning effect of participants after exercise is the best.

### 4.3. Temporal characteristics of the effects of acute moderate-intensity aerobic exercise on cognitive tasks

Two experiments showed that the exercise intervention group had significantly improved speed and accuracy 30 minutes after exercise compared to before exercise. The exercise intervention group also had significantly shorter reaction times 30 minutes after exercise than before in the first experiment, but the second experiment did not show a statistically significant difference. These results indicate that acute moderate-intensity aerobic exercise has temporal characteristics on cognitive tasks. Its effects can last up to 30 minutes after exercise, consistent with Peng and Zhou.^[19]^ The earliest theory about the relationship between cognitive function and acute aerobic exercise was proposed by Colin Davey in 1973 from the perspective of exercise awakening. He believed that exercise could awaken the autonomic nervous system while increasing brain metabolism and promoting cognitive processing efficiency.^[46]^ Moreover, studies have shown that moderate-intensity acute aerobic exercise positively affects the efficiency of attention resource allocation, can induce autonomic nervous system awakening, and bring it to its optimal awakened state. Additionally, after acute aerobic exercise, the concentration of BDNF increases significantly, which can enhance brain function, and the activity of BDNF can be maintained for up to 30 minutes after exercise. The increase in BDNF concentration helps the development of relevant cortices and neuroplasticity; the interaction between the 2 factors promotes cognitive performance.^[47]^ This experiment only found that the effects of exercise can be maintained for some indicators up to 30 minutes after exercise. This is because the experiment only observed the indicators 30 minutes after exercise and deeded to make more detailed observations after exercise. Some indicators may have maintained their effects but not for up to 30 minutes, so the effects were not observed then. Therefore, in future studies, the post-exercise indicator tests can be further subdivided to investigate how long the effects of exercise can be maintained.

In addition to the exercise awakening hypothesis and the neurotrophic factor BDNF, theories about acute aerobic exercise’s effects on cognitive performance include the frontal lobe dysfunction hypothesis, the neuroendocrine model, and the overcompensation theory of self-control strength.^[44]^ Both experiments have confirmed that acute aerobic exercise benefits cognitive performance. For e-sports players and other sedentary individuals, exercise intervention can improve cognitive performance, enhance their technical and operational abilities, and address the problem of sedentary behavior to ensure physical health. This research provides a reference for the future training and exercise rehabilitation of e-sports players.

### 4.4. Limitation

Our research results were observed only at the macroscopic level, focusing on the actual changes in various cognitive functions of E-athletes. The findings confirmed that acute aerobic exercise contributes to the improvement of certain cognitive functions in E-athletes, providing a basis for further research. However, 1 limitation of our study is that we did not further explain the cellular and molecular biological mechanisms at the micro level. Exercise can significantly increase the levels of biologically active molecules associated with improvements in cognitive function, including insulin-like growth factor 1, growth hormone, vascular endothelial growth factor, and brain-derived neurotrophic factor. Subsequent research could delve into the changes in the content of these relevant biologically active molecules, further elucidating their mechanisms at the cellular and molecular levels. Additionally, near-infrared spectroscopy could be employed to further observe the brain imaging results of E-athletes, thereby investigating the activating effects of acute aerobic exercise on different brain regions of E-athletes.

## 5. Conclusion

Acute moderate-intensity aerobic exercise significantly improves cognitive function in e-sports players and exhibits temporal characteristics. Its characteristics are mainly reflected in improving speed and accuracy, shortening visual reaction time, and improving instantaneous memory ability. The improvement effect can last 30 minutes after the end of the exercise. This suggests that e-sports players can adopt acute moderate-intensity aerobic exercise before competition or daily training to improve their performance.

## Author contributions

**Conceptualization:** Yuanyuan Song, Xinying Li.

**Data curation:** Weichao Zhang, Guoao Ma.

**Formal analysis:** Weichao Zhang.

**Funding acquisition:** Xiaoqiang Wang, Xun Li.

**Investigation:** Hongqiao Yan, Yuanyuan Song, Wenhua Zhang.

**Project administration:** Weichao Zhang, Xun Li.

**Resources:** Hongqiao Yan, Wenhua Zhang.

**Supervision:** Xinying Li.

**Validation:** Guoao Ma.

**Writing – original draft:** Weichao Zhang.

**Writing – review & editing:** Xiaoqiang Wang, Xun Li.
